# Levosulpiride for the treatment of diabetic macular oedema: a phase 2 randomized clinical trial

**DOI:** 10.1038/s41433-023-02715-5

**Published:** 2023-09-06

**Authors:** Carlos D. Núñez-Amaro, Mariana López, Elva Adán-Castro, Ma. Ludivina Robles-Osorio, Renata García-Franco, Marlon García-Roa, Yolanda Villalpando-Gómez, Paulina Ramírez-Neria, Nayeli Pineiro, Juan Fernando Rubio-Mijangos, Jorge Sánchez, Gabriela Ramírez-Hernández, Lourdes Siqueiros-Márquez, Nundehui Díaz-Lezama, Ellery López-Star, Thomas Bertsch, Gonzalo Marínez de la Escalera, Jakob Triebel, Carmen Clapp

**Affiliations:** 1 Facultad de Ciencias Naturales, Universidad Autónoma de Querétaro (UAQ), Querétaro, México; 2 Instituto Mexicano de Oftalmología (IMO), Querétaro, México; 3 Instituto de Neurobiología, Universidad Nacional Autónoma de México (UNAM), Campus UNAM-Juriquilla, Querétaro, México; 4 Instituto de la Retina del Bajío (INDEREB), Querétaro, México; 5 Institute for Clinical Chemistry, Laboratory Medicine and Transfusion Medicine, Nuremberg General Hospital and Paracelsus Medical University, Nuremberg, Germany

**Keywords:** Retinal diseases, Drug therapy, Outcomes research

## Abstract

**Background/Objective:**

The prokinetic levosulpiride elevates vasoinhibin levels in the vitreous of patients with proliferative diabetic retinopathy (PDR) suggesting clinical benefits due to the anti-vasopermeability and anti-angiogenic properties of vasoinhibin. We investigated the biological activity of levosulpiride in centre-involving diabetic macular oedema (DME).

**Patients/Methods:**

Prospective, randomized, double-blinded, dual-centre, phase 2 trial in patients with centre-involving DME orally treated with placebo (*n* = 17) or levosulpiride (*n* = 17) for 8 weeks or in patients with PDR undergoing elective pars plana vitrectomy and receiving placebo (*n* = 18) or levosulpiride (*n* = 18) orally for the 1 week before vitrectomy.

**Results:**

Levosulpiride improved changes from baseline in best-corrected visual acuity (*p* ≤ 0.037), central foveal thickness (CFT, *p* ≤ 0.013), and mean macular volume (MMV, *p* ≤ 0.002) at weeks 4, 6, and 8 compared to placebo. At 8 weeks, the proportion of eyes gaining ≥5 ETDRS letters at 4 m (41% vs. 28%), losing ≥21 μm in CFT (55% vs. 28%), and dropping ≥0.06 mm^3^ in MMV (65% vs. 29%) was higher after levosulpiride than placebo. The overall grading of visual and structural parameters improved with levosulpiride (*p* = 0.029). Levosulpiride reduced VEGF (*p* = 0.025) and PlGF (*p* = 0.008) levels in the vitreous of PDR patients. No significant adverse side-effects were detected.

**Conclusions:**

Oral levosulpiride for 8 weeks improved visual and structural outcomes in patients with centre-involving DME by mechanisms that may include intraocular upregulation of vasoinhibin and downregulation of VEGF and PlGF. Larger clinical trials evaluating long-term efficacy and safety are warranted.

## Introduction

Diabetic retinopathy (DR) and diabetic macular oedema (DME) are microvascular complications causing visual impairment in diabetes. Leakage from retinal capillaries in DME produces the accumulation of extracellular fluid and proteins that alter the structure and function of the macula and may lead to permanent loss of vision if untreated. Vascular endothelial growth factor (VEGF) isoforms, including placental growth factor (PlGF), are major vasopermeability factors in DME, and intravitreal agents blocking VEGF and PlGF have become a first-line of treatment for centre-involving DME with vision loss [1]. However, suboptimal responders and the invasiveness of frequent intravitreal injections have incentivized the development of new treatments [2].

Vasoinhibin, a fragment of the pituitary hormone prolactin (PRL), inhibits the permeability and growth of blood vessels [3, 4]. The generation of vasoinhibin depends on PRL levels and the activity of PRL-cleaving proteases that are regulated at the hypothalamus, the pituitary, and the target tissue levels defining the PRL/vasoinhibin axis [5]. This endocrine axis helps maintain corneal avascularity [6], restricts retinal vasculature [7], and is disrupted in retinopathy of prematurity [8, 9] and DR [10]. The intravitreal injection of vasoinhibin or its ocular overexpression reduces ischaemia-induced retinal angiogenesis [11] and prevents [12, 13] and reverses [14] diabetes- and VEGF-induced increase in retinal vasopermeability in rodents. Furthermore, the elevation of systemic PRL leads to the accumulation of vasoinhibin in the retina and inhibits the breakdown of the blood retinal barrier in diabetic rats [15]. These observations led to the hypothesis that medications causing hyperprolactinemia are beneficial in DME and DR and triggered a randomized phase 2 clinical trial in which levosulpiride-induced hyperprolactinemia is evaluated as a medical treatment in patients with DME and PDR [16].

Levosulpiride is a well-tolerated, dopamine D2 receptor blocker used as a prokinetic drug to treat diabetic gastroparesis [17]. At the pituitary level blockage of dopamine D2 receptors results in hyperprolactinemia [18]. The first partial outcome of the ongoing clinical trial provided a proof-of-concept by showing that levosulpiride increased PRL and vasoinhibin levels in the vitreous of patients with proliferative DR undergoing vitrectomy [19]. Further proof in diabetic rats showed that either racemic sulpiride or exogenous PRL increased ocular vasoinhibin and inhibited retinal hyper-vasopermeability [20]. Here, we describe the 2-month results of the clinical trial investigating the effect of levosulpiride in centre-involving DME.

## Patients and methods

This is a phase 2, prospective, double-blinded, randomized clinical trial conducted from May 2017 to November 2022 at two sites in Querétaro, México (Instituto Mexicano de Oftalmología and Instituto de la Retina del Bajío) and sponsored by the Consejo Nacional de Humanidades, Ciencias y Tecnologías (CONAHCYT, grants 247164, 289568, and A1-S-9620B) and the Universidad Nacional Autónoma de México (UNAM, grant 405PC). The study adhered to the guidelines of the Declaration of Helsinki, and the protocol and consent forms were approved by the Bioethics Committees of the Instituto de Neurobiología, UNAM and the Instituto Mexicano de Oftalmología. Each subject provided written informed consent and the study was supervised by an independent data and safety monitoring committee. The study was registered at www.clinicaltrials.gov in May 2017 under the identifier NCT03161652. The protocol details for the two study groups (DME and PDR patients undergoing vitrectomy), including settings and locations, eligibility criteria, enrollment and randomisation, blinding, outcome measures, sample size estimation, data collection and management, and safety, have been described previously [16].

### DME study

#### Protocol

From 176 screened mestizo patients with type 2 diabetes, there were 55 eligible centre-involving DME participants (aged 40–69 years, best-corrected visual acuity (BCVA) between 58 to 16 Early Treatment Diabetic Retinopathy Study (ETDRS) letters at 4 m (20/16 to 20/100 Snellen equivalent), and central foveal thickness (CFT) of ≥224 μm). After participants provided their written informed consent, blood samples were withdrawn to evaluate basal PRL levels and safety parameters [thyroid stimulating hormone (TSH), glycated haemoglobin (HbA1c), and creatinine]. Twelve patients were excluded [4 due to hyperprolactinemia (>20 ng/ml), 3 because of glomerular filtration rates <28 ml/min, 4 due to hard exudates in the fovea region, and 1 for receiving glaucoma treatment]. The remaining 43 subjects were randomized by the study coordinator based on a computer-generated list of random numbers in a 1:1 allocation ratio to receive levosulpiride (DISLEP®, Ferrer Therapeutics; 25-mg orally TID) or placebo (lactose pill orally TID) during a follow-up period of 8 weeks and study visits every 2 weeks. From the 43 subjects, 9 dropped out [5 did not comply with levosulpiride treatment confirmed by the circulating levels of PRL, 2 were hesitant to continue with study medication (one placebo and one levosulpiride), and 2 were unable to maintain visit appointments]. Thirty-four study subjects (12 females and 22 males) completed the study (17 treated with placebo and 17 with levosulpiride) and were evaluated [medical history, physical examination, BCVA, optical coherence tomography (OCT), blood and intraocular pressure, fundoscopy, and laboratory analysis of blood samples] at baseline and at study visits every 2 weeks. Determination of TSH was only done at baseline and HbA1c and fluorescein angiography were done at baseline and at week 8. Patients and investigators assessing outcomes were blind to treatment assignment.

#### Primary endpoints

BCVA, OCT, and fluorescein angiography were assessed by certified examiners using standardised protocols as reported [16]. Longitudinal changes (same eye before and after treatment) in BCVA, CFT, and mean macular volume (MMV) from baseline to weeks 2, 4, 6, and 8 were primary endpoints, as they reflect treatment impact over time. Assessments included the proportion of eyes at week 8 showing changes from baseline resulting in loss (−30 to −5), no change (−4 to +4), and gain (+5 to +15) of ETDRS letters at 4 m; improvement (−140 to −21 μm), no change (−20 to +20 μm), or worsening (+21 to +250 μm) of CFT; and improvement (−2.72 to −0.06 mm^3^), no modification (−0.05 to +0.05 mm^3^), or aggravation (+0.06 to +2.72 mm^3^) of MMV. Finally, an overall score of visual and anatomical outcomes at week 8 was provided by six independent retina specialists blinded to treatment certified by the Instituto Mexicano de Oftalmología Reading Centre. The overall change from baseline in all primary endpoints (BCVA, CFT, MMV, OCT macula image, fundoscopy, fluorescein angiography) was graded using a scale ranging from −4 to +4, in which −4 was highest worsening, 0 no change, and +4 highest improvement.

#### Secondary endpoint

PRL levels in serum confirmed adherence to levosulpiride treatment (also monitored by counting drug tablet return) and were quantified using the IMMULITE 2000 XPi immunoassay system (Siemens, Munich, Germany). The intra-assay and inter-assay coefficients of variation were less than 1%.

#### Safety assessment

Medical history, physical examination, ocular pressure, vital signs (sitting systolic and diastolic blood pressure), and laboratory tests in blood (HbA1c and creatinine) evaluated the safety of the study medication (levosulpiride vs. placebo). The occurrence of adverse effects was also sought by nondirective questioning of the patient at each visit or between visits.

### PDR study

#### Protocol

Briefly, from 175 screened PDR mestizo patients with type 2 diabetes undergoing elective primary pars plana vitrectomy, 41 eligible patients signed the informed consent, and their blood was withdrawn to evaluate PRL, HbA1c, and creatinine levels. From these 41 patients, 2 were excluded due to basal hyperprolactinemia (>20 ng/ml) and 3 because of vitrectomy being re-scheduled. All 36 patients (19 females and 17 males) met the inclusion criteria (aged 40–69 years, no history of prior vitrectomy, PRL serum levels ≤20 ng/ml, and glomerular filtration rate >28 ml/min) and were randomized 1:1 to receive placebo (orally TID, *n* = 18) and levosulpiride (25 mg orally TID, *n* = 18) for 1 week before vitrectomy. Blood samples were obtained immediately prior to surgery but before induction of anaesthesia to measure PRL levels. One millilitre of non-dilute vitreous was collected before fluid infusion using 25- or 27-gauge vitrectomy systems. Vitreous samples were stored at −80 °C until assayed for VEGF and PlGF levels. Twenty-nine vitreous samples were a subset of previously reported specimens [19].

#### Primary endpoints

VEGF and PlGF were measured in the same vitreous sample by enzyme-linked immunosorbent assay (ELISA) using the Quantikine Human VEGF and PlGF kits (R&D System, Minneapolis, MN) performed according to the manufacturer’s instructions. The intra-assay and inter-assay coefficients of variation for VEGF were 5.4% and 7.3%, respectively; and 5.4% and 11.2% for PlGF, respectively.

#### Secondary endpoint

PRL levels in serum at baseline and at vitrectomy were measured as indicated above.

### Statistics

GraphPad Prism Software Inc. version 6.01 was used. Statistical differences between two groups were determined by Student’s *t* test when their distribution was normal and the Mann–Whitney *U* test when it was not. The chi-square test was used to test differences between categorical variables. The threshold for significance was set at *p* < 0.05.

## Results

### DME patients’ demographics and clinical characteristics

Table 1 shows the demographics and clinical characteristics of the DME groups before and after treatment. The 34 patients with type 2 diabetes and centre-involving DME randomized to receive placebo (*n* = 17) or levosulpiride (*n* = 17) orally for 8 weeks were well balanced for baseline demographics (age, sex, body mass index, diabetes duration), clinical characteristics [HbA1c, kidney function (serum creatinine and glomerular filtration rate), blood and intraocular pressures], and serum PRL levels (Table 1). Both groups remained similar after 8 weeks of treatment, except for serum PRL values that significantly increased in patients receiving levosulpiride (Table 1).

**Table 1:** Demographic and clinical characteristics of the diabetic macula oedema groups.

Characteristic	Before treatment			After 8-week treatment		
	Placebo (*n* = 17)	Levosulpiride (*n* = 17)	*p	Placebo (*n* = 17)	Levosulpiride (*n* = 17)	*p
Age, years (SD)	61.0 (7.3)	58.5 (6.8)	0.32^a^			
Sex F *n* (%)	5 (29.4%)	7 (41.2%)	0.71^b^			
BMI (kg/m^2^)	27.1 (3.4)	27.0 (5.1)	0.95^a^			
DM2 years (SD)	16.9 (7.0)	19.4 (13.3)	0.66^c^			
HbA1c (SD)	8.8 (1.5)	8.1 (1.8)	0.27^a^	8.3 (1.7)	7.8 (1.8)	0.28^c^
SCr mg/dl (SD)	1.1 (0.3)	1.3 (0.4)	0.38^c^	1.2 (0.3)	1.3 (0.5)	0.59^c^
eGFR ml/min (SD)	65.3 (18.0)	56.9 (17.2)	0.17^a^	61.9 (14.3)	59.9 (28.2)	0.80^a^
BP mmHg (SD) Systolic	150.1 (20.1)	146.6 (21.0)	0.48^c^	145.9 (12.6)	153.4 (20.5)	0.40^c^
Diastolic	82.7 (10.9)	88.9 (17.2)	0.22^a^	87.6 (10.8)	94.6 (14.3)	0.12^a^
IOP mmHg (SD)	13.7 (2.4)	13.5 (2.6)	0.84^a^	14.4 (3.2)	13.1 (2.9)	0.17^a^
SPRL ng/ml (SD)	7.3 (2.4)	8.6 (3.6)	0.23^a^	7.7 (3.5)	150.1 (112.2)	0.00^c^

Values are means. Number of patients (*n*).

*SD* standard deviation, *BMI* body mass index, *DM2* diabetes mellitus type 2, *HbA1c* glycosylated haemoglobin, *sCr* serum creatinine, *eGFR* glomerular filtration rate (CKD-EPI equation), *BP* blood pressure, *IOP* intraocular pressure, *SPRL* serum prolactin.

*vs. respective placebo.

Based on ^a^Student’s *t* test, ^b^chi-square test, or ^c^Mann–Whitney *U* test.

The study eye characteristics (mean ± standard deviation) at baseline were comparable between placebo and levosulpiride groups in BCVA (37.3 ± 10.8 vs. 36.4 ± 8.7 number of ETDRS letters read at 4 m, *p* = 0.77) and MMV (8.5 ± 0.87 vs. 8.5 ± 1.2 mm^3^, *p* = 0.93). CFT values were slightly higher (325.9 ± 59.9 vs. 374.2 ± 89.5 μm, *p* = 0.06) in the group to receive levosulpiride.

### BCVA outcome

**Fig. 1:**
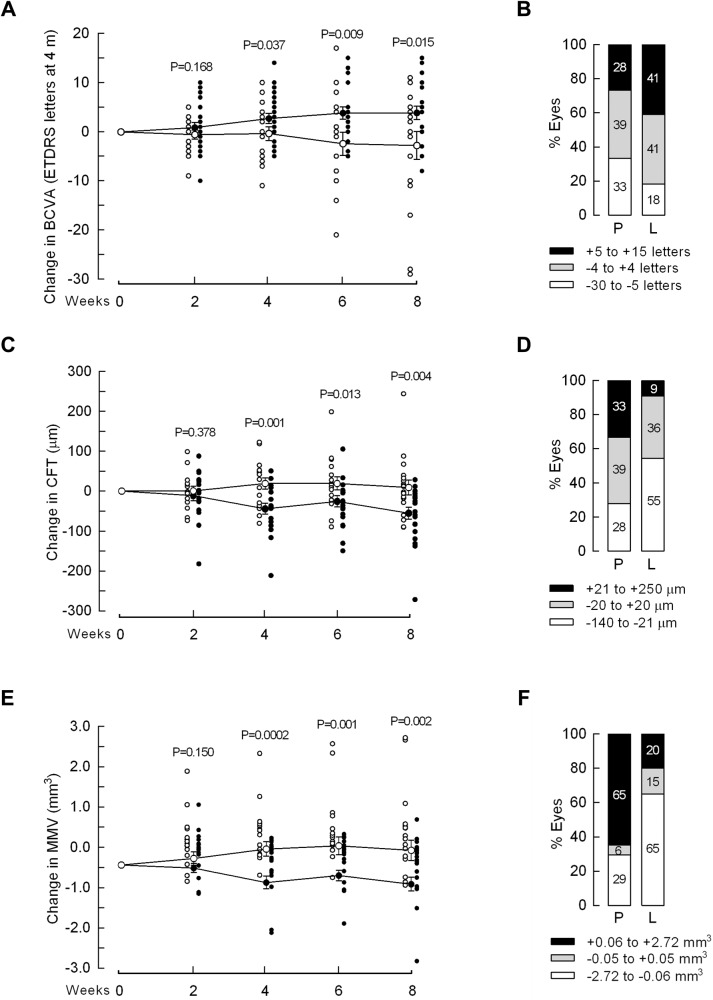
Levosulpiride improved the change from baseline in visual and structural parameters. Changes from baseline in best-corrected visual acuity (BCVA) (**A**), central foveal thickness (CFT) (**C**), and mean macula volume (MMV) (**E**) through 8 weeks of treatment with placebo (P, 18 eyes, white circles) or levosulpiride (L, 22 eyes, black circles). Mean ± SEM and individual values are indicated by big and small circles, respectively. Some individual values are identical and, thereby, hidden behind those shown. Comparisons (*t*-test) between same week L and P values are indicated. Proportion of eyes with different outcomes in BCVA (**B**), CFT (**D**), and MMV (**F**) after 8 weeks of treatment with P (18 eyes) or L (22 eyes).

As early as 4 weeks after initiating treatment with levosulpiride, the mean longitudinal change in BCVA over baseline was significantly higher than after placebo (Fig. 1A), with patients experiencing an average benefit of 6 ETDRS letters at week 8. At week 8 the percentage of eyes improving from baseline (+5 to +15 letters) was higher after levosulpiride than placebo (41% vs. 28%); the proportion of eyes with little change (−4 to +4 letters) was similar (41% vs. 39% in levosulpiride vs. placebo, respectively); and the percentage of eyes worsening (−30 to −5 letters) was lower after levosulpiride (18% vs. 33%) (Fig. 1B).

### CFT and MMV outcomes

The overtime BCVA improvement with levosulpiride was paralleled by longitudinal upgrades in CFT and MMV. The mean CFT longitudinal change of study eyes declined relative to baseline at weeks 4 to 8 after levosulpiride vs. placebo and accounted for a mean loss of 46.6 μm at the end of treatment (Fig. 1C). At week 8 the percentage of eyes improving from baseline (−140 to −21 μm) was higher after levosulpiride vs. placebo (55% vs. 28%); the proportion of eyes showing little change (−20 to +20 μm) was similar (36% vs. 39% for levosulpiride and placebo, respectively); and the percentage of eyes worsening (+21 to +250 μm) was lower after levosulpiride (9% vs. 33.3%) (Fig. 1D). Likewise, the mean change in MMV improved with levosulpiride. Starting at week 4 of levosulpiride treatment, the longitudinal change in MMV declined relative to baseline and resulted in the loss of 0.49 mm^3^ vs. placebo at week 8 (Fig. 1E). At week 8, the proportion of eyes improving (−2.72 to −0.06 mm^3^) in MMV from baseline was higher (65% vs. 29%) after levosulpiride; the percentage of eyes with small changes (−0.05 to +0.05 mm^3^) was similar (15% vs. 6%); and the proportion of eyes worsening (+0.06 to 2.72 mm^3^) was lower (20% vs. 65%) after levosulpiride (Fig. 1F).

### Overall visual and anatomic outcomes

**Fig. 2:**
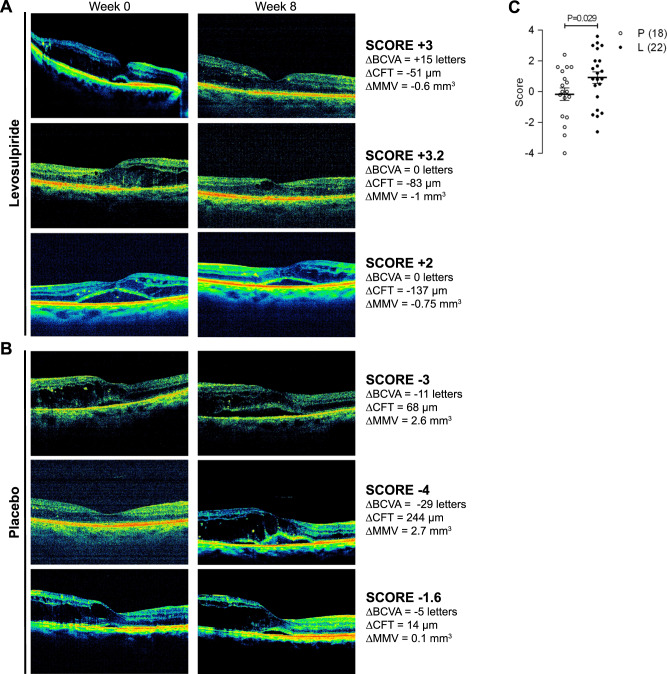
Levosulpiride improved the overall grading of primary endpoints. Three examples of levosulpiride- (**A**) and placebo- (**B**) treated patients showing the change from baseline (week 0) to week 8 of the same eye’s macular image, best-corrected visual acuity (ΔBCVA), central foveal thickness (ΔCFT), and mean macular volume (ΔMMV). Six independent retina specialists blinded to treatment scored the overall change in these and other (colour fundus photograph and fluorescein angiographic image) primary endpoints with a scale ranging from −4 to +4, where −4 was highest worsening, 0 no change, and +4 highest improvement. The score given to each example is indicated. **C** Given scores grading the overall change in all primary endpoints from baseline to week 8 of eyes from patients treated with placebo (P, 18 eyes) and levosulpiride (L, 22 eyes). Mean ± SEM and individual values are indicated. Comparison (*t*-test) between L and P scores.

Finally, the overall change from baseline to week 8 of all primary endpoints (BCVA, CFT, MMV, OCT macular image, fundoscopy, and fluorescein angiography) was evaluated by six independent retina specialists blinded to treatment through a scale that ranged from −4 to +4, where −4 was highest worsening, 0 no change, and +4 highest improvement. Figure 2 includes three examples of cases (A) and controls (B) showing the change from baseline (week 0) to week 8 in the same eye macular image, BCVA, CFT, and MMV, together with the overall score given to these and other (fundoscopy and fluoroangiography) primary endpoints. The mean score was higher (*p* = 0.029) for eyes from patients treated with levosulpiride than from the placebo group (Fig. 2C), supporting the overall improvement in visual and anatomical parameters by levosulpiride.

### Safety

Only two DME patients reported adverse effects. One patient in the placebo group informed tachycardia 2 weeks after treatment and abandoned the study, whereas one levosulpiride case reported somnolence, which could relate to the study medication as it is a known side effect of levosulpiride. Somnolence occurred 6 weeks after initiating treatment and the patient withdrew from the study. There were no significant differences in mean ocular pressure, mean serum levels of HbA1c, mean glomerular filtration rate, and mean blood pressure between the placebo- and levosulpiride-treated DME patients (Table 1).

### PDR patients’ demographics and clinical characteristics

The beneficial outcome of levosulpiride treatment was further investigated by a parallel arm of the clinical trial testing the effect of levosulpiride on the vitreous levels of VEGF and PlGF. The study consisted of 36 patients with type 2 diabetes and PDR undergoing elective pars plana vitrectomy due to vitreous haemorrhage or tractional retinal detachment. Patients were randomized to receive placebo (*n* = 18) or levosulpiride (*n* = 18) orally TID for 1 week before vitrectomy. Both groups were similar in age, sex, body mass index, diabetes duration, HbA1c, serum creatinine, glomerular filtration rate, and serum PRL levels at baseline (Table 2). As expected, PRL levels (mean ± standard deviation) increased significantly (146.5 ± 157.9 vs. 10.1 ± 5.9 ng/ml, *p* < 0.000) after levosulpiride, attesting adherence to levosulpiride treatment.

**Table 2:** Demographic and clinical characteristics of the proliferative diabetic retinopathy (PDR) groups at baseline.

	Placebo (*n* = 18)	Levosulpiride (*n* = 18)	*p*
Age years (SD)	57.6 (6.4)	56.2 (8.6)	0.58^a^
Sex F *n* (%)	10 (55.6)	9 (50)	0.73^b^
BMI (kg/m^2^) (SD)	28.2 (5.1)	28.5 (9.4)	0.42^c^
DM2 years (SD)	15.8 (5.9)	16.0 (6.3)	0.92^a^
HbA1c, % (SD)	7.5 (1.4)	8.0 (1.9)	0.42^a^
SCr mg/dl (SD)	1.2 (0.5)	1.0 (0.4)	0.27^c^
eGFR ml/min) (SD)	69.9 (24.8)	83.8 (27.4)	0.12^a^
SPRL ng/ml (SD)	7.7 (4.0)	10.3 (5.7)	0.10^c^

All patients were with PDR before undergoing elective pars plana vitrectomy and oral treatment with placebo or levosulpiride. Values are means. Number of patients (*n*).

*BMI* body mass index, *DM2* type 2 diabetes mellitus, *HbA1c* glycosylated haemoglobin, *SCr* serum creatinine, *eGFR* estimated glomerular filtration rate (CKD-EPI equation), *SPRL* serum PRL.

*p* values vs. placebo based on Students *t* test^a^, chi-square test^b^, and Mann–Whitney *U* test^c^.

### Vitreous levels of VEGF and PlGF

**Fig. 3:**
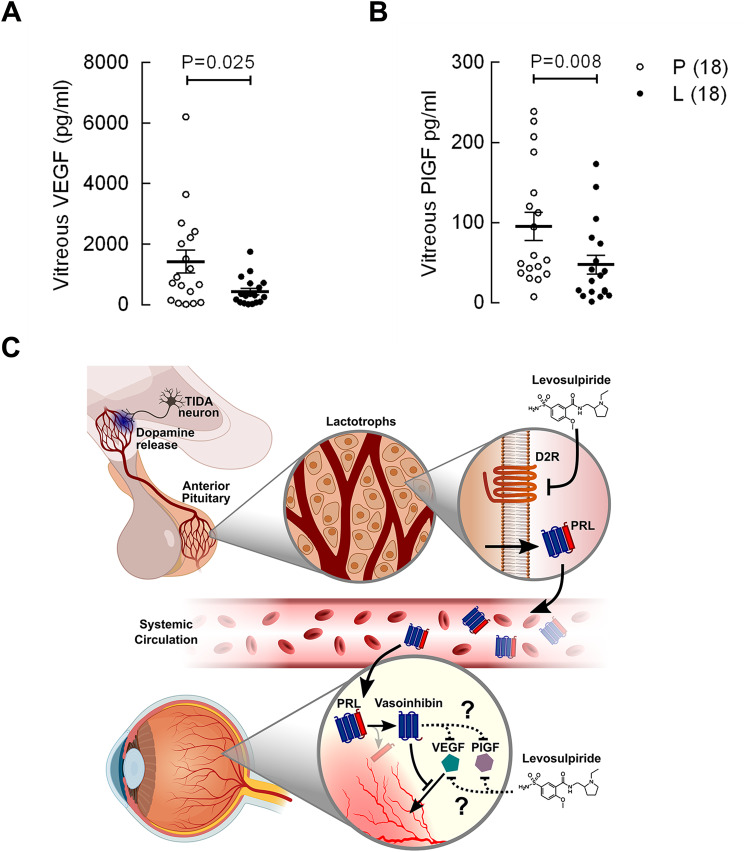
Levosulpiride reduced the vitreous levels of VEGF and PlGF by mechanisms that may involve the prolactin/vasoinhibin axis. Vascular endothelial growth factor (VEGF) (**A**) and placental growth factor (PlGF) (**B**) concentrations in the vitreous of patients with proliferative diabetic retinopathy (PDR) undergoing vitrectomy and treatment with placebo (P, 18 patients) or levosulpiride (L, 18 patients). Mean ± SEM and individual values are shown. Comparisons (*U* test) between L and P. **C** Schematic representation of the mechanisms by which levosulpiride therapy could limit the progression of DME and DR. Levosulpiride is a dopamine D2 receptor (D2R) antagonist that induces hyperprolactinemia by blocking dopamine receptors in the membrane of pituitary lactotrophs mediating the inhibition of prolactin (PRL) release. Hyperprolactinemia favours the intraocular incorporation of PRL and its proteolytic conversion to vasoinhibin, which, in turn, inhibits VEGF-induced permeability and growth of blood vessels. In addition, levosulpiride downregulates VEGF and PlGF intraocular levels by mechanisms that may or may not depend on the PRL/vasoinhibin axis. This is a modified version of the original figure published by Nuñez-Amaro et al. [19] under the Creative Commons Attribution-Non-Commercial-No Derivatives 4.0 International License (https://creativecommons.org/licenses/by-nc-nd/4.0/).

The vitreous concentrations of VEGF and PlGF were significantly reduced in PDR patients treated with levosulpiride (Fig. 3A and B).

## Discussion

Levosulpiride is a prokinetic [17] that induces hyperprolactinemia as a side effect [18]. We have reported that levosulpiride increases the levels of the PRL fragment, vasoinhibin, in the vitreous of patients with PDR [19]. Because vasoinhibin is a potent inhibitor of retinal hyper-vasopermeability in rodent models of DME [12–14], we hypothesised a beneficial outcome of levosulpiride in DME patients [16, 19]. As proof of principle, we showed that either racemic sulpiride or exogenous PRL elevate ocular vasoinhibin and inhibit the increase in retinal vasopermeability in diabetic rats [20]. Here, we report that the oral administration of levosulpiride improves visual and structural outcomes in patients with centre-involving DME by mechanisms that may also include the downregulation of VEGF and PlGF intraocular levels.

The study was designed to assess the efficacy of levosulpiride in previously untreated DME patients with the caveat of deferring for the end of the study any other treatment. Accordingly, the protocol was short-termed (8 weeks) and carried out in DME patients with moderate vision loss (defined as ≥16 ETDRS letters at 4 m; Snellen equivalent ~20/100) that were carefully evaluated every 2 weeks. Levosulpiride showed meaningful responses compared with the natural history of the process (demonstrated by the placebo group). Mean changes in visual and anatomical parameters improved early (4 weeks) after initiating levosulpiride treatment. At week 8, gain and losses from baseline in BCVA (+6 letters), CFT (−46.6 μm), and MMV (−0.49 mm^3^) were comparable to those reported after 4 weeks of intravitreal ranibizumab (gain of ~6 letters and loss of 92 μm in CFT) [21] and after 12 weeks of ranibizumab plus prompt/deferred laser (average gain of ~7 letters) [22]. Furthermore, the higher scores given to longitudinal changes in all ophthalmologic parameters (BCVA, CFT, MMV, OCT macula image, fundoscopy, and fluorescein angiography) confirmed the overall benefit of levosulpiride treatment.

Levosulpiride efficacy rate in gain of vision (41%) was lower than efficacy rates in CFT (55%) and MMV (65%). Improvements in anatomical parameters are indicative of resolution of macular oedema and may [21] or may not [22] associate with visual acuity benefits. Lack of correlation is not unexpected since retinal oedema is not the only factor affecting reduced visual acuity. Anatomic improvements occur after the selective blockage of members of the VEGF family [1, 2] and suggest an anti-vasopermeability action of levosulpiride at the macular level. This effect could be accomplished by the increase in intraocular vasoinhibin induced by levosulpiride [19, 20], since vasoinhibin inhibits the excessive retinal vasopermeability in response to VEGF and the diabetic condition [12–14]. Furthermore, we now show that levosulpiride reduces the levels of VEGF and PlGF in the vitreous of PDR patients. The vitreous levels of both growth factors were like those previously reported in active PDR [23, 24] and their reduction by levosulpiride was statistically significant. This effect contrasts with that of an anti-VEGF monoclonal antibody (bevacizumab) that reduces VEGF but not PlGF in the vitreous [24], whereas both VEGF and PlGF are targeted by the soluble decoy receptor aflibercept, a property that may explain the higher therapeutic efficacy of this medication in DME [1, 25]. The mechanism mediating the inhibitory effect of levosulpiride on VEGF and PlGF vitreous levels is unknown, but could operate through vasoinhibin since vasoinhibin reduces VEGF expression in arthritic joints [26]. Because VEGF and PlGF promote the development and progression of DME [2, 27, 28], their inhibition by levosulpiride provides further confidence that the observed efficacy of levosulpiride treatment is reliable and meaningful.

Beneficial outcomes are outstanding when considering that levosulpiride is a non-invasive orally active medication that contrasts with the standard care of DME using intravitreal drug delivery, which poses a risk of ocular complications [29]. In fact, due to their invasiveness and high cost, intravitreal anti-VEGF medications are not recommended for patients with centre-involving DME and good vision in the setting of proper follow-up [30]. Important concerns remain, however, that absence of treatment is doing too little to prevent subsequent vision loss in these patients [31]. Levosulpiride offers the opportunity of an early intervention against the worsening of DME even in patients with good vision. Its oral route favours compliance and dosage particularly in the setting of clinical practice, where rates of patient follow-up and administration of anti-VEGF therapies may be lower than in clinical trials [32].

As expected, levosulpiride did not pose any significant safety concern during the 8 weeks of treatment. We used the oral dose of levosulpiride (25 mg TID) employed for the treatment of diabetic gastroparesis, a complication found in 5% of diabetic patients [33]. This dose is well-tolerated during short (2–16 weeks) and long (4–42 months) administrations [17, 34–36]. For example, after 4 weeks of levosulpiride treatment, a multicentre study in 342 dyspeptic female and male patients reported 40 cases (11%) with adverse effects (26% galactorrhoea, 17% somnolence, 11% fatigue, and 11.5% headache), none of whom abandoned the study [34]. Also, a 6-month study in 40 patients with diabetes type 1 and gastroparesis showed adverse effects (breast tenderness, loss of libido, and/or drowsiness) in 2 and 1 patients with levosulpiride and placebo, respectively [35]. Nonetheless, hyperprolactinemia-associated alterations would considerably limit the potential for long-term clinical use in women with pre-existing menstrual irregularities, men with sexual dysfunction, women who are trying to conceive, or persons with PRL-related tumours (breast cancer and pituitary tumours) [37]. It should also be mentioned that women appear to be more prone to the adverse manifestations of levosulpiride-induced hyperprolactinemia than men [18]. This may relate to the fact that levosulpiride elevates circulating PRL to higher levels in women than in men [19] and/or to menstrual abnormalities and galactorrhoea being usually quite obvious in females and thereby diagnosed more frequently.

This study had several strengths including a powerful assessment of adherence to treatment monitored by the high efficacy of levosulpiride-induced hyperprolactinemia [37]; a placebo group referencing the natural progress of the disease; participants and clinicians masked to treatment assignment; and visit schedules that were strictly followed according to the protocol. Among the study limitations stand the use of vitreous samples from PDR and not DME patients due to lack of access to vitreous specimens in the latter; a small number of patients, although low *p* values substantiate major conclusions; the short duration of the study, limiting efficacy comparisons with most studies that involve longer periods of time. Finally, we acknowledge that our conclusions are based on data generated from a rigorously conducted clinical trial and, thereby, may vary in a clinical setting where patients may be subjected to an inconsistent follow-up.

In summary, this interventional, randomized, placebo-controlled phase 2 study, in which levosulpiride was administered orally according to indications of use in daily practice, showed the effectiveness and safety of this prokinetic agent for improving visual and structural outcomes in patients with centre-involving DME. Its mechanism of action may involve the PRL/vasoinhibin axis [5]. Levosulpiride is a dopamine D2 receptor antagonist that induces hyperprolactinemia by blocking dopamine receptors in the pituitary lactotrophs causing inhibition of PRL release (Fig. 3C). Hyperprolactinemia favours the intraocular incorporation of PRL and its proteolytic conversion to vasoinhibin [19, 20] which, in turn, can lead to a reduction in VEGF-induced increase in retinal vasopermeability [12–14, 20] (Fig. 3C). Moreover, levosulpiride downregulates VEGF and PlGF intraocular levels by mechanisms that may or may not depend on the PRL/vasoinhibin axis (Fig. 3C).

Our study repositions levosulpiride outside its prokinetic scope as a safe, affordable, and early treatment for DME, DR, and other vascular retinopathies. Larger and longer studies are needed to solidify these findings.

## Summary

### What was known before

- Elevation of systemic prolactin leads to the intraocular accumulation of vasoinhibin, a proteolytic fragment of prolactin that inhibits the excessive permeability and growth of blood vessels in the retina.
- The prokinetic oral medication, levosulpiride, induces hyperprolactinemia and elevates the levels of vasoinhibin in the retina of diabetic rats and in the vitreous of patients with proliferative diabetic retinopathy.
- Levosulpiride may have beneficial outcomes in diabetic macular oedema and diabetic retinopathy due to the vascular properties of vasoinhibin.

### What this study adds

- Oral levosulpiride for 8 weeks improves visual and structural outcomes in patients with centre-involving diabetic macular oedema by mechanisms that may include the upregulation of vasoinhibin and the downregulation of VEGF and PlGF intraocular levels.
- No significant adverse side-effects were detected.
- Our study repositions levosulpiride outside its prokinetic scope as a non-invasive, affordable, early treatment for diabetic macular oedema, diabetic retinopathy, and other vascular retinopathies.
- Larger and longer studies are needed to solidify these findings.

## Data Availability

All original data generated or analysed during this study are included in this published article.
